# Minimally Invasive Sacroiliac Joint Fusion: 2-Year Radiographic and Clinical Outcomes with a Principles-Based SIJ Fusion System

**DOI:** 10.2174/1874325001812010007

**Published:** 2018-01-17

**Authors:** William W Cross, Arnold Delbridge, Donald Hales, Louis C Fielding

**Affiliations:** 1 Mayo Clinic Rochester, Minnesota, MN 55905, United States; 2 Cedar Valley Medical Specialists PC Waterloo, Iowa, IA 50704, United States; 3 Northern Arizona Orthopaedics Flagstaff, Arizona, AZ 86001, United States; 4 Tahoe Labs, LLC 1057 Montgomery St., San Carlos, California, 94070, United States

**Keywords:** Sacroiliac joint, Sacroiliac fusion, Decortication, Minimally invasive, Fusion, Radiographic fusion, Bridging bone, Low back pain

## Abstract

**Background:**

Sacroiliac joint (SIJ) degeneration is a common source of low back pain (LBP). Minimally invasive (MI) SIJ fusion procedures have demonstrated meaningful clinical improvement. A recently developed MI SIJ fusion system incorporates decortication, placement of bone graft and fixation with threaded implants (DC/BG/TF).

**Patients and Methods:**

Nineteen patients who had MI SIJ fusion with DC/BG/TF were enrolled at three centers. Fusion was assessed in CT images obtained 12 and 24 months postoperatively by an independent radiographic core laboratory. LBP was assessed using a 0-10 numerical pain scale (NPS) preoperatively and at 12 and 24 months postoperatively.

**Results:**

At 12 months, 15/19 patients (79%) had bridging bone across the SIJ, and at 24 months 17/18 patients (94%) available for follow-up had SIJ fusion. Of the patients with bridging bone 88% had fusion within the decorticated area, with solid fusion in 83%. A significant reduction in NPS scores was demonstrated, representing a 73% reduction in average low back pain.

**Conclusion:**

The patients in this series demonstrated significant improvement in LBP. Fusion rates at 24 months demonstrate promise for this system, which utilizes the established orthopedic principles of DC/BG/TF to achieve arthrodesis. Further study is warranted to demonstrate comparative fusion rates for different implant systems.

## 1 INTRODUCTION

Pain originating in the sacroiliac joint (SIJ) is increasingly recognized as a common and significant source of low back pain (LBP), representing a debilitating condition with a great cost to society [1]. While the sources and etiology of LBP are often indeterminate and controversial, it has been shown that degenerative SIJ pain may account for as much as 15-30% of patients with axial LBP [2-5]. While conservative measures may provide some relief, fusion of the SIJ is indicated when nonoperative care has failed to provide persistent improvement. In the past decade, minimally invasive (MI) SIJ fusion techniques have become the preferred intervention, having shown superior outcomes to both open SIJ fusion and nonoperative care [6-8].

With the increasing recognition of SIJ disease, MI SIJ fusion systems have proliferated [9-13]. Studies of these systems have shown promising clinical and economic value [12, 14, 15]. While the goal of MI SIJ fusion surgery is to promote fusion leading to long-term pain relief, radiographic fusion rates are not often nor well reported. Few studies report fusion outcomes, but those that do vary from 25%-90% of patients at varying time points of 6 months to 5 years for different technologies [12, 14, 16-18]. Within these studies various imaging modalities are used and only some utilize independent radiographic reviewers. The variability in radiographic fusion assessment among studies of MI SIJ fusion does not provide conclusive demonstration of the rate and timing of the development of arthrodesis with these technologies.

An MI SIJ fusion system has been developed that incorporates decortication, bone grafting and fixation with threaded implants. The combination of decortication, graft placement and fixation with threaded implants is postulated to promote fusion of the SIJ more often and earlier than previously reported by other systems. This study prospectively assessed radiographic fusion status and improvement in low back pain at 12 and 24 months for patients treated with MI SIJ fusion including decortication and bone grafting (clinicaltrials.gov identifier NCT02425631).

## 2 PATIENTS AND METHODS

### 2.1 Patient Selection

Nineteen patients at three centers (Mayo Clinic, Rochester, MN; Cedar Valley Medical Specialists, Waterloo, IA; Northern Arizona Orthopaedics, Flagstaff, AZ) were enrolled in this study. All patients who had undergone an MI SIJ fusion procedure with decortication, bone grafting and threaded implant fixation within the previous 12 months were recruited for the study. There were no exclusion criteria, as all patients, regardless of their medical history, were deemed appropriate. Willing participants provided informed consent and agreed to comply with study procedures, including CT scans at 12 and 24 months postoperatively. The study protocol was approved by each investigational site’s IRB.

## 3 SURGICAL INTERVENTION

### 3.1 Sacroiliac Joint Fusion System

The sacroiliac joint fusion system utilized in this study (SImmetry Sacroiliac Joint Fusion System, Zyga Technology, Minnetonka, MN) has been described previously [10, 11]. Briefly, the system uses a proprietary decortication instrument to prepare approximately 5cm^2^ of the ilial and sacral bone surfaces, removing cartilage and allowing placement of approximately 5cc of bone graft. A 12.5 mm threaded implant is placed through the prepared fusion bed (primary device), and an optional, secondary 6.5mm threaded implant provides additional mechanical stability during the fusion process if the surgeon considers additional fixation appropriate based upon patient anatomical and available bone stock considerations. The system is illustrated in Fig. (**1**).

### 3.2 Surgical Technique

Patients are placed prone on a radiolucent flat top table. Chest bolsters are placed to elevate the pelvis and assist with image acquisition. The posterior buttocks and proximal thighs are prepped into the sterile field. Pelvic inlet and outlet views are utilized to obtain an appropriate starting point into the preoperatively templated trajectory based on sacral anatomy. Most often, this trajectory resided in the S1 vertebral body. Once this starting point is verified on both pelvic views with fluoroscopy, a 3.2mm guide pin is placed across the ilium and across the SI joint. Dilators are used and a working portal inserted.

A 9mm drill is then used to drill across the ilium and across, but not through the SI joint. The working cannula is then malleted into the ilium, over the drill, to maintain the correct trajectory across the SI joint. The drill is then removed and autograft collected from the drill flutes for later bone grafting into the fusion zone. A flat ended scraper is utilized to remove cartilage from the sacral side of the SI joint. Three sequential, flexible, deployable decorticating curettes are then used to prepare the joint surfaces for arthrodesis. Each of these decorticators is progressively more aggressive. Osteochondral debris is irrigated and removed from the fusion area and autologous bone graft from the drill flutes as well as, optionally, a bone graft extender such as demineralized bone matrix (DBM) (*e.g*., Grafton, Medtronic, Minneapolis, MN) are then applied through the working cannula. The guide pin is reinserted into the center position of the working portal and placed into the appropriate trajectory in the sacrum. Minor corrections can be made at this point to ensure safe passage into the sacrum. The 9mm drill is used again to drill across the SI joint into the sacrum. The length is measured and the primary implant is placed across SI joint. The implant is verified to be fully seated by using an anterior-posterior fluoroscopic view with 20 to 30 degrees of ‘rollback’ allowing the surgeon to visualize the outer table of the ilium.

A 6.5mm secondary, anti-rotation implant is then applied using a cannulated system in most cases. This screw is placed using the same fluoroscopic views and is inserted either directly cranial or caudal to the main implant based on preoperative planning and sacral morphology. No joint decortication is used in the application of this anti-rotation screw. The single incision is copiously irrigated and local anesthetic applied followed by a layered soft-tissue closure. Typical post-operative instructions include 2-3 weeks of partial weight bearing followed by auto-advancing patients as tolerated. No deep vein thrombosis prophylaxis was utilized.

## 4 OUTCOME ASSESSMENTS AND STATISTICAL TECHNIQUES

### 4.1 Radiographic Fusion Assessment

Axial CT images with coronal, paracoronal and sagittal reconstructions were obtained 12 and 24 months postoperatively. All unformatted digital images were transferred to an independent radiographic core laboratory (Medical Metrics Inc., Houston, TX) for analysis. Qualitative and quantitative assessments were performed by two independent radiographic evaluators and an adjudicator. The evaluators were blinded to each other’s assessments, and disagreement between the two primary evaluators were resolved by the adjudicator. All evaluators were board-certified, fellowship-trained, practicing musculoskeletal radiologists with no financial interest in the study sponsor. The radiographic evaluators were trained on the schedule of assessments, classification system for recording each assessment, the radiographic features of the device and its design. They did not have access to clinical outcomes data and the same evaluators were assigned to the study for its duration. The radiographic fusion definitions are described in Table **1**. In addition to fusion status, the core laboratory determined the location of fusion and proximity to implanted devices. The radiographic core laboratory has determined that for these techniques of assessment of radiographic SIJ fusion, Gwet’s AC1 chance-corrected agreement coefficient between the two primary reviewers was 0.67 for bridging relative to anatomy, 0.67 for bridging relative to the primary device, and 0.63 for bridging relative to the secondary device [19]. Using the Landis and Koch criteria for judging kappa statistics, the agreement would be considered substantial [20].

### 4.2 Clinical Assessment

Low back pain (LBP) was assessed preoperatively and postoperatively at 12 and 24 months with a 0-10 numerical pain scale (NPS). Overall patient satisfaction with the implant system and smoking status were collected at both the 12 and 24 month visits. Any untoward medical occurrence that comprised a negative change from baseline was considered an adverse event (AE), and required to be promptly reported to the sponsor. AE assessment included classification of the AE as serious or non-serious, review of device-related AEs and any additional procedures performed or required since the procedure. A minimal clinically important difference (MCID) was defined as an NPS improvement of at least 2/10 [21].

### 4.3 Statistical Analysis

Changes in low back pain were assessed using 1-tailed Student’s t-test. Paired t-tests were used for same-subject data. A significance level of p≤0.05 was used. Confidence intervals for parametric statistics were calculated assuming a t-distribution. Binomial confidence intervals were calculated using the normal approximation method.

## 5 RESULTS

### 5.1 Patient Demographics

Nineteen patients who had undergone MI SIJ fusion with decortication and bone grafting were enrolled at three centers. All nineteen subjects were available for 12 month follow-up. One patient withdrew from the study after the 12 month visit; the remaining 18 patients were available for the 24 month follow-up. Patient demographics are summarized in Table **2**.

### 5.2 Surgical Procedure

All surgical procedures were successfully completed as intended, with no intraoperative complications reported. All procedures were performed unilaterally. Fourteen (74%) patients had both primary and secondary threaded fixation implants; five (26%) had only a single threaded fixation implant. The number of implants utilized was at the surgeons discretion based upon patient anatomy and perceived safety issues with placing another implant. There were no statistically significant differences in the outcomes in patients treated with 1 or 2 implants. It is the authors opinion that a single implant placed with compression can provide significant stability given the tongue-in-groove orientation of most sacral ala. The bone grafting procedure was uniform across the three investigators, and consisted of approximately 3-5 cc of autograft from iliac crest drilling as part of the procedure as well as 5cc of demineralized bone matrix. Additional surgical procedure data are summarized in Table **3**.

### 5.3 Radiographic Fusion Results

**Fig. (2):**
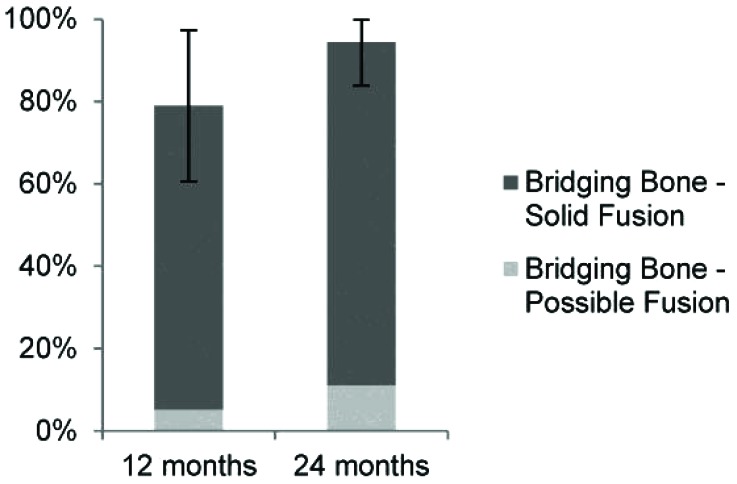
Radiographic fusion status (error bars represent 95% CI).

Fusion status, as assessed and adjudicated by the independent radiographic core laboratory, is summarized in (Fig. **2** and Table **4**). Examples of CT images assessed as bridging bone with solid fusion, bridging bone with possible fusion and not fused are shown in Fig. (**3**). Fifteen of 19 (79%) were assessed to have demonstrated radiographic fusion at 12 months follow-up, and 17/18 (94%) at 24 months follow-up. Of patients exhibiting bridging bone, 14/15 (93%) at 12 months and 15/17 (88%) at 24 months were assessed as having solid fusion. The fusion location was found to be within the decorticated region around the primary implant in 87% and 88% of subjects exhibiting bridging bone (13/15 at 12 months; 15/17 at 24 months).

Of the four patients that had not yet demonstrated fusion at 12 months follow-up, by 24 months one had demonstrated solid fusion, one demonstrated possible fusion, one was not fused, and one was lost to follow-up. Fusion status was compared to baseline characteristic, and was found to have no association with smoking status, gender, age or BMI.

### 5.4 Low Back Pain

**Fig. (4):**
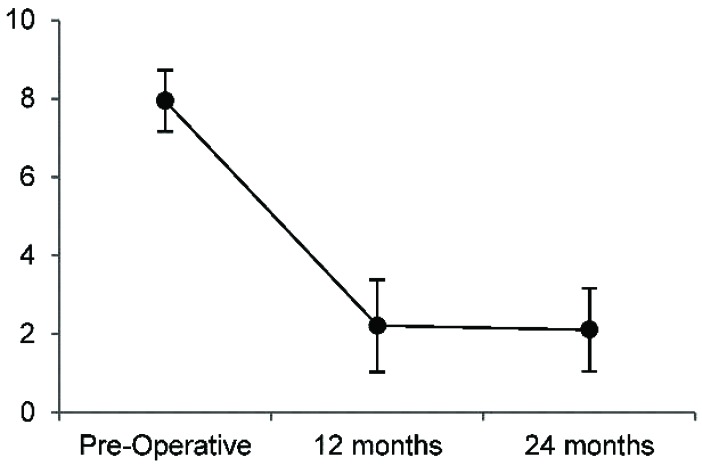
Low Back Pain (numerical pain scale, 0-10; error bars represent 95% CI).

LBP results are summarized in Fig. **4** and Table **5**. Mean preoperative LBP was 7.9/10, and reduced to 2.2/10 12 months postoperatively and 2.1/10 24 months postoperatively, representing a 72% and 73% reduction in LBP, respectively. The reduction in LBP were significant (p<0.01) at both 12 and 24 months, with large effect sizes of -3.5 and -2.9, respectively. At 12 months, 19/19 (100%) of patients met the MCID of ≥2/10 improvement on the NPS; at 24 months follow-up 17/18 (94%) met the MCID.

### 5.5 Adverse Events and Patient Satisfaction

There were no procedural complications, nor were any device or procedure-related serious adverse events (SAEs) reported at any time point. At 12 and 24 months respectively, four (21%) and two (11%) patients had experienced device-related adverse events, however as mentioned previously, none of these were classified as SAEs. At 24 months, 17/18 (94%) were satisfied with their result, and 16/18 (89%) would recommend the surgery to another.

## 6 DISCUSSION

In the current study, following principles of orthopedic fusion surgery, radiographically proven arthrodesis was achieved in over 90% of our cohort; 79% and 94% of patients demonstrated bridging bone 12 and 24 months, respectively, after MI SIJ fusion surgery that included decortication, bone graft and threaded fixation. Patients reported clinically significant and durable improvements in pain, with 75% reduction at 24 months follow-up, comparable to the improvements seen in other reports of MI SIJ fusion and superior to reported results for nonoperative care [14, 17, 22].

We feel our results are the product of the accepted orthopedic principles of arthrodesis, including decortication, autograft and secure fixation. Decortication exposes subcortical blood and marrow, which provide vascularization, mesenchymal stem cells, osteogenic and inflammatory factors that promote the bone growth necessary for fusion [23, 24]. Tissue differentiation of skeletal stem cells is determined by both the loading environment and vascularity [25, 26]. Insufficient fixation resulting in excessive residual joint motion may create local tensile loads in the remodeling tissue. This mechanical environment would result in stem cell differentiation into fibrous connective tissue. Conversely, even with proper fixation, without the vascularity provided by decortication, local hydrostatic pressure would induce tissue differentiation into cartilage [25, 26]. In addition to being a generally accepted orthopedic principle, this hypothesis is supported by recent work by Spain *et al* where SIJ screw fixation without decortication or grafting resulted in higher reoperation rates [27]. Similarly, Duhon *et al* reported bone adherent or adjacent to >30% of the surface area of >90% of triangular titanium implants at 12 months follow-up; however bridging bone across the SIJ representing true fusion formation was only seen in 25% of cases and 48.3% of patients in the same study continuing to take opioids at 24 months follow-up [12, 22]. In these cases, it would seem that mechanical fixation was provided without the vascular and biological environment necessary for a true arthrodesis. In the present study, 88% of fusions at 24 months were through the decorticated region, supporting this mechanism of action.

**Fig. (5):**
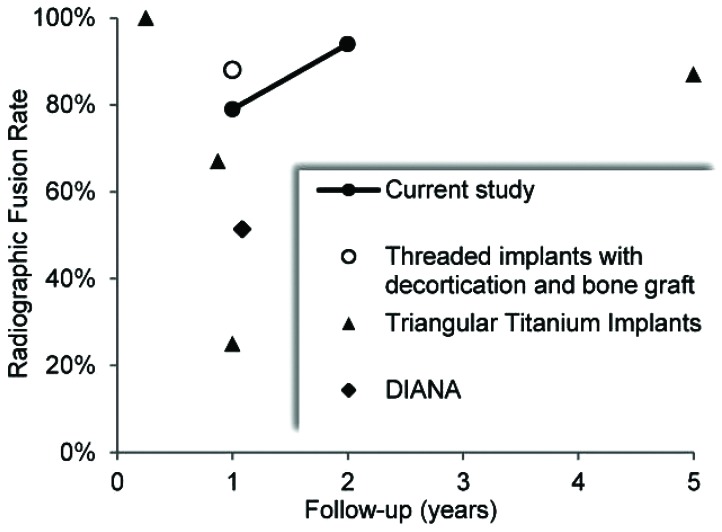
Comparison of current and reported radiographic fusion results for MI SIJ fusion [12-14, 16-18, 31].

It is notable that fusion rates with MI SIJ fusion systems are not well reported in the literature. This is stark contrast to other studies in the spine regarding lumbar and lumbosacral fusions where radiographic arthrodesis is actively sought and documented to help demonstrate success [28, 29]. In a systematic review of MI SIJ fusion by Heiney *et al,* only two of 18 included studies reported fusion rates [30]. While radiographic fusion outcomes of MI SIJ procedures are rarely and inconsistently reported, the results of this study compare favorably to the fusion rates reported for various MI SIJ fusion technologies, as summarized in Fig. **5** and Table **6**. At approximately 1 year postoperatively, reported fusion rates for triangular titanium implants ranged from 25% to 67%, increasing to 87% at 5 years, while a 51.4% fusion rate was reported for the distraction interference arthrodesis neurovascular anticipating (DIANA) system [12, 14, 18, 31]. The high fusion rate reported by Rudolf *et al* at 5 years follow-up likely reflects the longer process of auto-arthrodesis resulting from joint immobilization, rather than the fusion mass that develops with decortication and bone graft.

Weaknesses in our study include the small patient sample size. Importantly, it was not powered to detect associations between radiographic fusion status and clinical outcomes and instead, the primary outcome assessment was radiographic fusion. The paucity of reported fusion rates vs. clinical outcomes demands further investigation. Larger prospective, comparative studies should enable predictive association of preoperative variables, fusion status and implant system characteristics to clinical outcomes. Strengths of our study include the use of independent, non-biased musculoskeletal radiologists as reviewers. Previous studies have used the authors and industry consultants to provide radiographic fusion data which can clearly bias the interpretation of radiographic fusion. Our study included both radiographic assessments and clinical outcomes up to 24 months. These data show continued improvements with both metrics indicating that improvements in patient outcomes can be expected to continue beyond the one-year mark.

In summary, the clinical improvements seen with MI SIJ fusion systems, particularly when compared to nonoperative care, are clearly evident with this and other studies and cannot be disputed. The radiographic fusion results seen in this series are further encouraging that consistently durable results can be achieved in appropriately selected patients.

## CONCLUSION

Patients treated with MI SIJ fusion utilizing decortication, bone grafting, and implantation of threaded implants demonstrated significant improvement in low back pain. Fusion rates at 24 months as assessed in CT imaging, including evidence of bridging bone in 94% of patients and solid fusion extending from the ilium to sacrum in 83% demonstrate promise for this system, which utilizes the established orthopedic principles of decortication, bone grafting and threaded fixation to achieve arthrodesis. Further, study is warranted to demonstrate comparative fusion rates for different implant systems and predictive correlation to clinical outcomes.

## Figures and Tables

**Fig. (1):**
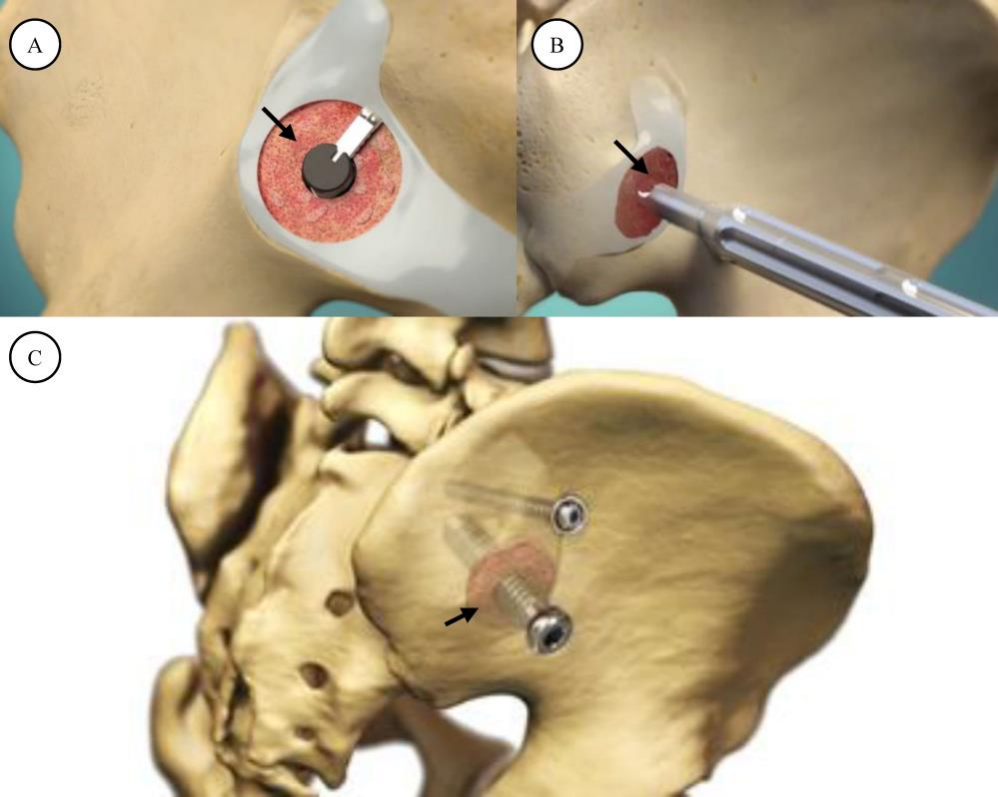
SImmetry System. Panel A illustrates the decorticator instrument preparing the graft bed (arrow); the decorticated region is then packed with approximately 5cc of graft material (B; arrow). Final implant construct (C), showing a 12.5mm cannulated implant (inferior) with surrounding decorticated area and graft (arrow) and 6.5mm anti-rotation implant (superior).

**Fig. (3):**
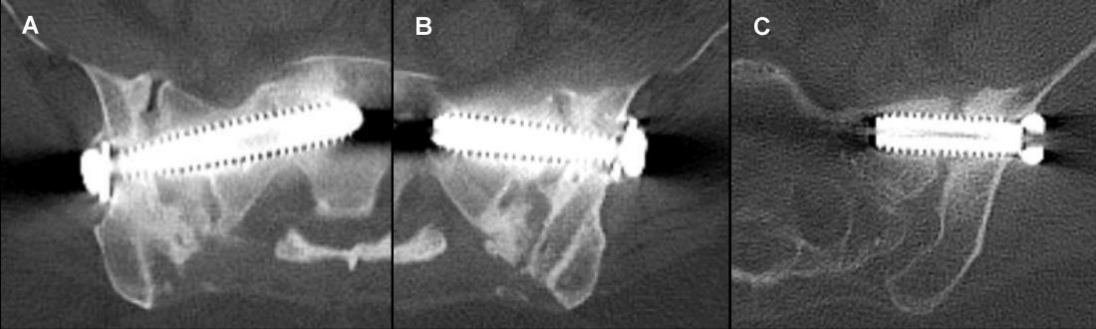
CT images assessed as bridging bone with solid fusion (A), bridging bone with possible fusion (B) and not fused (C).

**Table 1:** Radiographic fusion assessment.

Solid Fusion	Presence of solid continuous bridging across the treated joint
Possible Fusion	Presence of possible continuous bridging across the treated joint
No Fusion	No bridging bone
Ind - Indeterminate	A reliable determination cannot be made from the available imaging due to technical factors, sub-optimal image quality, obscured anatomy, obstructed view or other imaging artifacts.
UA - Unable to assess	The relevant images are missing or unavailable for review, or the relevant anatomy is not visible in the field of view.

**Table 2:** Summary of patient demographics.

Female	15/19 (79%)
Age (years) Mean ± SD Range	60.1 ± 13.7 30.8 – 84.4
BMI (kg/m^2^) Mean ± SD Range	29.1 ± 5.7 21.0 – 41.3
Smoking at time of visit 12 months 24 months	3/19 (16%) 0/18 (0%)

**Table 3:** Surgical procedure data.

Unilateral procedure –	19/19 (100%)
Treated side Left Right	7/19 (37%) 12/19 (63%)
Fixation with threaded implants Primary and Secondary (two implants) Primary only (one implant)	14/19 (74%) 5/19 (26%)
Duration (minutes) Mean ± SD Range	83.5 ± 29.0 35.0 – 135.0
Estimated Blood Loss (ml) Mean ± SD Range	57.1 ± 44.9 0.0 – 125.0

**Table 4:** Radiographic fusion status.

–	**12 Months**	**24 Months**
N	19	18
**Adjudicated Fusion Status**	–	–
Bridging bone present 95% CI	15/19 (79%) 61% - 97%	17/18 (94%) 84% - 100%
Solid fusion	14/19 (74%)	15/18 (83%)
Possible fusion	1/19 (5%)	2/18 (11%)
No fusion	4/19 (21%)	1/18 (6%)
**Fusion location**	–	–
Fusion within the decortication area of the primary device	13/15 (87%)	15/17 (88%)

**Table 5:** Low back pain results.

–	**Preoperative**	**12 months**	**24 months**
N	19	19	18
**Low Back Pain (NPS, 0-10)**			
Mean±SD	7.9 ± 1.6	2.2 ± 2.4	2.1 ± 2.1
Range	6 – 10	0 – 7	0 – 7
95% CI	7.2 – 8.7	1.0 – 3.4	1.0 – 3.2
**Postoperative Improvement in Low Back Pain (paired data)**			
–	Mean±SD	5.7 ± 1.7 (76% ± 25%)	5.7 ± 2.0 (75% ± 27%)
–	95% CI	4.9 - 6.5 (63% - 88%)	4.7 - 6.7 (61% - 88%)
–	p	<0.01	<0.01
–	Effect Size	-3.5	-2.9

**Table 6:** Current and reported bridging bone fusion rates for MI SIJ fusion.

**Study**	**N**	**Fusion Rate**	**Follow-up**	**Notes**
Current study	19 18	79% 94%	12 months 24 months	CT imaging; independently assessed and adjudicated for bridging bone. Threaded implant fixation with decortication and graft placement.
Kube *et al* [17]	20	88%	12 months	Thin slice (<2mm) CT imaging assessment of bony bridging across the SIJ and absence of lucency. Threaded implant fixation with decortication and graft placement.
Duhon *et al* [12]	159	25%	12 months	CT imaging assessment of bridging bone either adjacent or distant to triangular titanium implants.
Rudolf *et al* [14]	15	87%	5 years	CT imaging assessment of evidence of osseous bridging across the SIJ. Triangular titanium implants.
Treon *et al* [18]	37	51%	13 months	CT assessment of fusion status after distraction interference arthrodesis neurovascular anticipating (DIANA) posterior MI SIJ fusion.
Gaetani *et al* [13]	12	100% (“initial fusion”)	3 months	CT imaging demonstrated “initial fusion” at 3 months with triangular titanium implants.
Schroeder *et al* [27]	6	67%	4-15 months	Successful fusion defined as bridging bone across the SIJ in an axial or coronal CT scan or on two x-ray views. Triangular titanium implants.
